# The impact of multiple representations on students' understanding of vector field concepts: Implementation of simulations and sketching activities into lecture-based recitations in undergraduate physics

**DOI:** 10.3389/fpsyg.2025.1544764

**Published:** 2025-04-25

**Authors:** Larissa Hahn, Pascal Klein

**Affiliations:** Physics Education Research, Faculty of Physics, University of Göttingen, Göttingen, Germany

**Keywords:** multiple representations, task-based learning, lecture-based recitations, sketching, interactive visualization, conceptual understanding, physics, simulation

## Abstract

Multiple external representations (e. g. diagrams, equations) and their interpretations play a central role in science and science learning as research has shown that they can substantially facilitate the learning and understanding of science concepts. Therefore, multiple and particularly visual representations are a core element of university physics. In electrodynamics, which students encounter already at the beginning of their studies, vector fields are a central representation typically used in two forms: the algebraic representation as a formula and the visual representation depicted by a vector field diagram. While the former is valuable for quantitative calculations, vector field diagrams are beneficial for showing many properties of a field at a glance. However, benefiting from the mutual complementarity of both representations requires representational competencies aiming at referring different representations to each other. Yet, previous study results revealed several student problems particularly regarding the conceptual understanding of vector calculus concepts. Against this background, we have developed research-based, multi-representational learning tasks that focus on the visual interpretation of vector field diagrams aiming at enhancing a broad, mathematical as well as conceptual, understanding of vector calculus concepts. Following current trends in education research and considering cognitive psychology, the tasks incorporate sketching activities and interactive (computer-based) simulations to enhance multi-representational learning. In this article, we assess the impact of the learning tasks in a field study by implementing them into lecture-based recitations in a first-year electrodynamics course at the University of Goettingen. For this, a within- and between-subjects design is used comparing a multi-representational intervention group (IG) and a control group (CG) working on traditional calculation-based tasks (*N* = 81). Group comparisons revealed that students in the intervention group scored significantly higher on a vector field performance test after the intervention (*p* = 0.04, *d* = 0.40) while perceiving higher cognitive load during task processing (extraneous *p* < 0.001, *d* = 0.75; intrinsic *p* = 0.02, *d* = 0.47; germane *p* = 0.02, *d* = 0.48). Moreover, students who worked with multi-representational learning tasks achieved higher normalized learning gains in tasks addressing conceptual understanding and representational competencies related to vector field diagrams and vector calculus concepts (*g*_*H, IG*_ = 0.35, *g*_*H, CG*_ = 0.13). These results provide guidance for the design of multi-representational learning tasks in field-related physics topics and beyond.

## 1 Introduction

Mathematics and physics concepts are often represented in some form of external representation (De Cock, 2012). Thereby, different forms of representation, multiple representations (MRs), allow to express a concept or a (learning) subject in various manners by focusing on different properties and characteristics. In complementing and constraining each other, multiple representations enable a deep understanding of a situation or a construct (Ainsworth, 1999; Seufert, 2003) and, moreover, using multiple representations was found to have positive effects on knowledge acquisition and problem-solving skills (e.g., Nieminen et al., 2012; Rau, 2017). Regarding the understanding and communication of science concepts, visual representations are particularly crucial (Cook, 2006). Following previous research, they can help to eliminate science concepts' abstract nature and were shown to support students to develop scientific conceptions (e.g., Cook, 2006; Chiu and Linn, 2014; Suyatna et al., 2017). However, to benefit from multimedia learning environments, representational competencies based on an understanding of how individual representations depict information, how they relate to each other, and how to choose an appropriate representation to solve a problem are required (DeFT framework; Ainsworth, 2006). Without representational competencies, visual representations cannot fully unfold their potential as meaning-making tools.

Additionally, learning with and mentally processing visual representations often places special demands on the visuo-spatial working memory, thus increasing cognitive load (Baddeley, 1986; Cook, 2006; Logie, 2014). Here, previous research showed that externalizing visuo-spatial information can provide cognitive relief (e.g., Bilda and Gero, 2005). In this regard, sketching (or drawing) visual cues in multimedia learning has become an increasing scientific focus in recent years (Ainsworth and Scheiter, 2021). Following empirical findings, sketching allows to pay more attention to details (Ainsworth and Scheiter, 2021), thus supporting a visual understanding of concepts (Wu and Rau, 2018). Correspondingly, previous studies reported positive learning effects of sketching activities in (multi-)representational learning environments, as they increase attention and engagement with the representations and help to activate prior knowledge, to understand a representations' properties, or to recall information (e.g., Ainsworth and Scheiter, 2021; Kohnle et al., 2020; Leopold and Leutner, 2012; Wu and Rau, 2018). Typical sketching activities are copying a given representation, creating a visual representation with modified individual features or by transforming textual information into a drawing, or inventing a novel representation (e.g., to reason; Ainsworth and Scheiter, 2021; Kohnle et al., 2020). Moreover, with respect to Cognitive Load Theory (Sweller, 2010) which characterizes the limited capacity of working memory resources based on three types of cognitive load—intrinsic, extraneous, and germane cognitive load—sketching activities are able to promote a more effective use of these resources (Bilda and Gero, 2005).

In addition to cognitive relief provided by sketching in multi-representational learning, previous work demonstrated the added value of interactive (computer-based) simulations for the development of representational competencies (e.g., Kohnle and Passante, 2017; Stieff, 2011). As such, integration of such visualization tools in multimedia learning environments foster active learning, thus supporting students' use of scientific representations for communication and helping them to integrate their representational knowledge systematically with content knowledge (Linn et al., 2006; Stieff, 2011). Specifically, the complementation of simulation-based learning by the aforementioned sketching activities was found to support a deeper understanding of the representation being presented (Ainsworth and Scheiter, 2021; Kohnle et al., 2020; Wu and Rau, 2018).

Considering the value of multiple representations for science learning, unsurprisingly, they also play a major role in university physics. For instance, in electrodynamics, vector field representations are deeply rooted in the developmental history of the domain (Faraday, 1852), being represented either algebraically as a formula or graphically using arrows. In university experimental lectures, an introduction to electric and magnetic fields typically starts from concrete analogous representations of electric or magnetic field lines, then moving on to more abstract or idealized visual-graphical and symbolical representations (Küchemann et al., 2021). Using demonstration experiments, electric and magnetic field lines are visualized, for example, by semolina grains (Benimoff, 2006; Küchemann et al., 2021; Lincoln, 2017) or iron filings (Küchemann et al., 2021; Thompson, 1878), respectively. When representing a quantity as a vector field, the fields' properties, its divergence and curl, and further the integral theorems of Gauss and Stokes are of particular importance for physics applications (Griffiths, 2013). Accordingly, a sound understanding of vector calculus is of great importance for undergraduate and graduate physics studies. For example, a study by Burkholder et al. (2021) found a significant correlation between extensive preparation in vector calculus and students' performance in an introductory course on electromagnetism.

However, further research also revealed that a conceptual understanding, which is relevant to physics comprehension, often caused difficulties for students (e.g., Bollen et al., 2015; Pepper et al., 2012; Singh and Maries, 2013). Besides conceptual gaps regarding vector field representations in general, learning difficulties in dealing with vector field concepts such as divergence and curl became particularly apparent. For example, students struggled to extract information about divergence or curl from vector field diagrams and they interpreted and used these concepts intuitively instead of referring to their physics-mathematical concepts in a rigorous manner (Ambrose, 2004; Baily et al., 2016; Bollen et al., 2015, 2016, 2018; Klein et al., 2018, 2019; Pepper et al., 2012; Singh and Maries, 2013). In a study on students' difficulties regarding the curl of vector fields, Jung and Lee (2012) diagnosed the gap between mathematical and conceptual reasoning as a major source of comprehension problems. Furthermore, Singh and Maries (2013) concluded that graduate students struggle with the concepts of divergence and curl, even though they know how to calculate them mathematically. In the context of electrostatics and electromagnetism, it was also shown that conceptual gaps regarding vector calculus led to improper understanding and errors when applying essential principles in physics (Ambrose, 2004; Bollen et al., 2015, 2016; Jung and Lee, 2012; Li and Singh, 2017). Regarding these findings, it is noticeable that the aforementioned studies did not strictly distinct between conceptual understanding and representational competencies with respect to vector fields. This is not surprising, since there is strong overlap of the two areas in electrodynamics—vector fields are, as such, a form of representation that cannot be understood in a subject context isolated from concepts. Conversely, it is almost impossible to learn electrodynamics concepts without vector field representations (representational dilemma; Rau, 2017).

In introductory physics texts, vector concepts are typically given as mathematical expressions, but are either not or insufficiently explained qualitatively (Smith, 2014). Even in more advanced physics textbooks, there is little geometric explanation or discussion of vector field concepts and integral theorems. Regarding the aforementioned empirical findings, relevance and requirement of new instructions that address a conceptual understanding become even more apparent. Consequently, numerous authors advocated the use of visual representations in order to foster a conceptual understanding. Following this line of research, Bollen et al. (2018) developed a guided-inquiry teaching-learning sequence on vector calculus in electrodynamics aiming at strengthening the connection between visual and algebraic representations. Implementing the tutorials in a second-year undergraduate electrodynamics course revealed a positive effect of the interventions on physics students' conceptual understanding and their ability to visually interpret vector field diagrams. In addition, subjects expressed primarily positive feedback regarding the learning approach. However, as discussed by the authors, the exact results should be interpreted with care as the number of participants was small and the implementation followed a less streamlined structure as, for example, no strict control and intervention group design was used. Additionally, Klein et al. (2018, 2019) developed text-based instructions for visually interpreting divergence using vector field diagrams. Eye tracking was used to analyze representation-specific visual behaviors, such as evaluating vectors along coordinate directions. Here, gaze analyses revealed an increase in conceptual understanding as a result of this intervention (Klein et al., 2018, 2019). In addition to a positive impact of visual cues on performance measures, a positive correlation with students' response confidence was found. This means that students not only answered correctly more often, but also trusted their answers more, which is a desirable result of successful teaching (Klein et al., 2017, 2019; Lindsey and Nagel, 2015). In subsequent interviews, subjects expressed diagram-specific mental operations, such as decomposing vectors and evaluating field components along coordinate directions, as a main problem source (Klein et al., 2018). Thus, a follow-up experimental study involved sketching activities aiming at generating representation-specific aids (e.g., field components) to support the visual interpretation of divergence (Hahn and Klein, 2023a). Here, sketching was shown to significantly reduce perceived (intrinsic) cognitive load when applying visual problem-solving strategies related to a fields' divergence.

With regard to previous findings concerning student problems, building upon the existing multi-representational teaching-learning materials, and using the DeFT framework (Ainsworth, 2006), four multi-representational learning tasks were developed aiming at visually interpreting vector field diagrams (see Hahn and Klein, 2022b, for task development). Each task addresses one vector calculus concept in which the representational forms are used in a coordinated manner aiming at developing conceptual understanding (Modeling Instruction approach, e.g., McPadden and Brewe, 2017). Using a combined approach, multiple representational learning was integrated with sketching activities and an interactive vector field visualization tool (Hahn et al., 2024), following current trends in education research (Ainsworth and Scheiter, 2021; Kohnle et al., 2020; Wu and Rau, 2018). Sketching activities and the interactive tool were incorporated to provide cognitive relief in multi-representational learning, to foster engagement with the representations, and to support the development of representational competencies related to vector calculus concepts. Here, representation-specific sketching activities, such as sketching vector components or highlighting rows or columns to support evaluation along coordinate directions, were included (Hahn and Klein, 2023a; Klein et al., 2018, 2019). Additionally, typical sketching tasks for learning with interactive simulations, such as copying or creating a vector field diagram, were involved (Kohnle et al., 2020). The research-based multi-representational, sketching- and simulation-based learning tasks (in the following: multi-representational learning tasks) are implemented into lecture-based recitations in a first-year electrodynamics course. Consequently, the present study aims at evaluating the added value of a combined approach including multiple representations, sketching activities, and interactive visualizations in task-based learning of vector calculus by comparing a multi-representational intervention group and a control group with traditional calculation-based tasks. Therefore, the following guiding question is investigated: “Do multi-representational learning tasks have a higher learning impact than traditional (calculation-based) tasks in the context of vector fields?" Considering previous research findings and theoretical frameworks from cognitive psychology on multi-representational learning, and on the use of sketching activities and interactive visualizations, we hypothesize that multi-representational, sketching- and simulation-based learning tasks (in the following: multi-representational learning tasks):

**(H1)** promote students' performance as measured by a vector field performance test (that includes tasks related to vector calculus, vector field quantities, and vector field concepts), and
**(H2)** reduce perceived cognitive load (as measured by a cognitive load questionnaire) during task processing.

## 2 Methods

Learning tasks are implemented in the weekly recitations on *experimental physics II* in the summer semester 2022, 2023, and 2024. Physics students usually attend *experimental physics II* in their second semester of study, then encountering university electromagnetism for the first time. The module includes a lecture with demonstration experiments and weekly recitations in which the compulsory assignments are discussed. Dividing the study into an alpha implementation (summer semester 2022) and a beta implementation (summer semester 2023 and 2024) primarily serves to consolidate the data. In the alpha implementation, all instruments and learning tasks were tested and psychometrically characterized, thus providing guidance for improvement (see Hahn and Klein, 2023b, for results of the alpha implementation). Then, alpha as well as beta implementation are used to evaluate the effectiveness of the intervention aiming at answering the guiding question and testing the hypotheses. Study design and procedure are identical in both implementations as there where no fundamental changes necessary after the alpha implementation. In the following, the study and the materials used are described (Section 2.1). Then, in Section 2.2, sample and statistical methods used aiming at answering the guiding question and testing the hypotheses are presented.

### 2.1 Study design and materials

A detailed description of the study procedure, the materials and all instruments can be found in Hahn and Klein (2023b). The article also describes test and scale analyses based on data from the alpha implementation.

#### 2.1.1 Procedure

**Figure 1:**
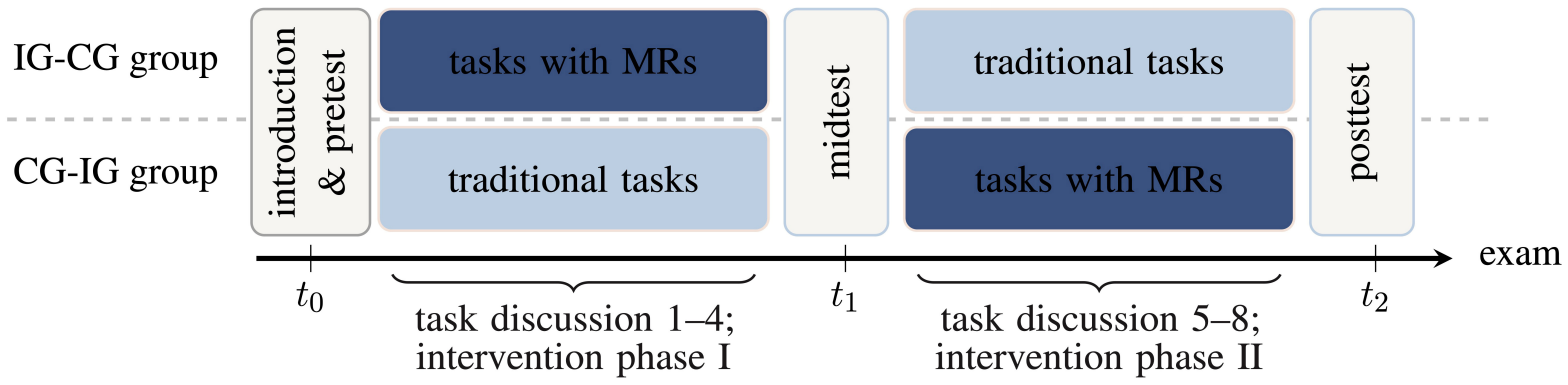
Study design with timeline from left (*t*_0_) to right (*t*_2_; intervention group IG, control group CG, multiple representations MRs). The designations “IG-CG group" and “CG-IG group" refer to the chronological order of the groups in the rotational design (first intervention group, then control group, or vice versa).

The study is based on within- and between-subjects treatments wrapped in a rotational design (Figure 1). At the beginning of the lecture period, all recitation groups are randomly divided into two superordinate groups both serving as intervention groups (IG) and control groups (CG) at some time but in different order (IG-CG group and CG-IG group, respectively). Students select a fixed recitation group by their own without knowing about the assignment to a treatment condition later on. The study procedure including an overview of all instruments and data is summarized in Table 1 (see Section 2.1.2 and results of the alpha implementation in Hahn and Klein, 2023b). Before the first intervention phase (intervention phase I), students take a performance test on vector calculus. Subsequently, the first intervention phase starts and in each of the following 4 weeks, students complete a mandatory intervention task (either a multi-representational or a traditional task) in addition to a set of standard tasks which does not differ between the groups. The traditional tasks consist of typical, predominantly calculation- and formula-based, problem-solving tasks that have always been used in the course (e.g., they present some mathematical representation of vector fields and students must calculate divergence or curl). First, the upper group in Figure 1 is intervention group (IG) and works on the multi-representational learning tasks, while the lower group is control group (CG) and works on traditional (calculation-based) tasks. All assignments are completed by self-study within 1 week, submitted for correction, and discussed with a dedicated, independent intervention tutor during the subsequent recitation. Prior to each task discussion, a short questionnaire on perceived cognitive load during task processing and means of task assistance is deployed. After the first intervention phase in the 7th week of the semester, students again complete the performance test on vector calculus and another evaluation questionnaire. Subsequently, the groups switch roles and the second 4-week intervention phase starts. Finally, the performance test on vector calculus and the questionnaire are administered again.

**Table 1:** Overview of scales included for data analysis (Cronbach's alpha α_*C*_).

**Time point**	**Scale**	**#item**	**α_*C*_**
*t*_0_ (pretest)	Vector field performance test (V_0_) (response accuracy)	44	0.76
	Confidence (C_0_)	34	0.96
	Sociodemographics	–	–
Intervention phase I	Extraneous cognitive load (ECL)	16	0.87
	Intrinsic cognitive load (ICL)	12	0.90
	Germane cognitive load (GCL)	8	0.88
	Effort (E)	8	0.82
*t*_1_ (posttest)	Vector field performance test (V_1_) (response accuracy)	44	0.71
	Confidence (C_1_)	34	0.97
	Tutor (T)	6	0.82

#### 2.1.2 Materials and measures

*Vector field performance test*. Initially, all subjects completed a test with demographic questions (e.g., age, gender, semester of study) and a performance test on vector calculus assessing conceptual understanding closely linked with representational competencies. The performance test included 19 tasks, partly comprising several subtasks, hence, a total of 65 items (multiple-choice and true-false items of one task counted separately) covering seven different subtopics of vector calculus. Forty-niner of the items were designed in multiple-choice or true-false format, while the remaining 16 items required a sketch, formula, justification, calculation, or a proof. Most of the items were taken from established concept tests on electrodynamics (CURrENT) or have been used and validated in a similar form in previous studies (Baily et al., 2016, 2017; Bollen et al., 2015, 2018; Hahn and Klein, 2022a, 2023a; Klein et al., 2018, 2019, 2021; Rabe et al., 2022). In Table 2, two of these tasks are specifically referred to in order to characterize the sample: one targets foundational knowledge (i.e., basic principles of vector field representations), while the other emphasizes the transfer of knowledge (i.e., applying it in novel contexts or problem-solving scenarios, such as curl evaluation). After the intervention phases, the students again completed the performance test which was extended by a module-specific task on electrostatics. Additionally, for most of the multiple-choice and true-false items, response confidence was assessed using a 6-point Likert-type rating scale (1 = absolutely confident to 6 = not confident at all) to provide insight into student response behavior beyond performance measures.

**Table 2:** Sample data (intervention group IG, control group CG, number No., statistical significance *p* using unpaired two-sided *t*-tests).

	**Total**	**IG**	**CG**	***p***
		**(IG-CG group)**	**(CG-IG group)**	
Number of subjects	81	51	30	–
No. of female subjects	18 (22%)	10 (20%)	8 (27%)	–
Mean age (in years)	20.3 ± 2.8	20.3 ± 1.8	20.3 ± 4.0	0.91
No. of semesters studied	2.3 ± 1.2	2.2 ± 1.0	2.4 ± 1.4	0.41
Average grade for university entrance^a^	1.5 ± 0.5	1.5 ± 0.5	1.4 ± 0.5	0.57
Tutor behavior (T)^b^	0.85 ± 0.14	0.84 ± 0.15	0.87 ± 0.12	0.28
Pretest score vector field performance test (V_0_)^b^	0.69 ± 0.14	0.68 ± 0.14	0.71 ± 0.15	0.30
Pretest score vector field representations^b, c^	0.81 ± 0.18	0.80 ± 0.19	0.84 ± 0.18	0.25
Score for qualitative evaluation of a field's curl^b, c^	0.31 ± 0.41	0.28 ± 0.38	0.38 ± 0.46	0.31

^a^The scale ranges from 1.0 (best performance) to 4.0. The grades are indicated by the students.

^b^The scale ranges from 0 to 1 (best performance).

^c^Item is included in the vector field performance test.

*Questionnaire on task processing*. In weekly recitations, students answered a short questionnaire related to the previous learning task providing information about the cognitive load they experienced while completing the task as well as any kind of task assistance. The items regarding cognitive load are based on a scale measuring the three types of cognitive load from Leppink et al. (2013) which was supplemented by items from Klepsch et al. (2017) and Krell (2017). The final questionnaire contained 12 items measuring cognitive load on a 6-point Likert-type rating scale (1 = strongly disagree to 6 = strongly agree). Test analyses in the alpha implementation resulted in four scales of cognitive load (Hahn and Klein, 2023b). The scales for extraneous, intrinsic, and germane cognitive load reflect the three types of cognitive load according to Sweller (2010), with the germane cognitive load scale primarily assessing perceived improvement in understanding, the intrinsic cognitive load scale addressing the inherent complexity of the learning subject, and the extraneous cognitive load scale focusing on the design of the instructional material. In addition, the effort scale assesses the effort expended in task completion (Krell, 2017; Paas and Van Merriënboer, 1994). In addition to the perceived cognitive load, means of task assistance (e.g., “working together in a group with students from my course,” “looking up in a textbook”) were assessed using a choice format. This information was used to maximize the comparability between the two groups and to ensure that students are actively involved in the learning process.

*Questionnaire on tutor behavior*. After the intervention phases, a questionnaire was used which surveyed the tutor's behavior during task discussion as a control variable using six items (6-point Likert-type rating scale from 1 = strongly disagree to 6 = strongly agree). The items are based on the “tutor evaluation questionnaire” by Dolmans et al. (1994) supplemented by modifications from Baroffio et al. (1999) and Pinto et al. (2001).

*Learning tasks*. The multi-representational learning tasks were designed as four parallel learning tasks on divergence, Gauss' theorem, curl, and Stokes' theorem building on the sketching-based instruction for divergence developed by Hahn and Klein (2023a) (see Hahn and Klein, 2022b, for task development). These tasks integrate multiple representations and sketching activities, further supported by the inclusion of an interactive vector field visualization tool (Hahn et al., 2024). In the learning tasks, interactive visualizations and sketching activities are closely linked, for example, students are required to create a vector field diagram based on the tool. In line with common practice in university teaching, the control groups' tasks primarily involve calculations and mathematical proofs in the context of vector calculus. Multi-representational as well as traditional calculation-based learning tasks can be found in the Supplementary material.

### 2.2 Analyses of alpha and beta implementation

Due to high dropout rates, the core sample, which includes students who participated in all performance tests and completed all eight learning tasks, would consist of only 10 students (*N*_IG_ = 6, *N*_CG_ = 4), which contradicts fundamental assumptions of statistical data analysis. Therefore, the following analyses, aimed at answering the guiding question and testing the hypotheses, will be limited to intervention phase I, including 81 students. Since the rotational design was primarily implemented for fairness reasons, the research aim is not compromised by omitting intervention phase II. A summary of deviations in data analysis, as proposed in Hahn and Klein (2023b), is provided at the end of the manuscript.

#### 2.2.1 Sample

In total, 281 students took part in the pretest. However, to ensure valid results by focusing on intervention phase I, data analysis will be carried out based on a sample of 81 student who participated in both the pre (*t*_0_) and post (*t*_1_) intervention vector field performance test and completed all four learning tasks of intervention phase I (for detailed description of sample generation see Hahn and Klein, in press). This sample size is consistent with the power analysis results from the alpha implementation (for further characterization of the sample, see Table 2; Hahn and Klein, 2023b). It is notable that the pretest scores on vector field representations, the first item of the performance test, were rather high, indicating that all students had sufficient prior knowledge of visual representations of vector fields and the decomposition of individual vectors into components to understand the learning tasks. However, since they scored only 69% in the pretest on vector calculus and as only 31% were able to evaluate the curl of a vector field diagram (item included in the performance test; detailed analysis of this item in Hahn and Klein, in press), the tasks could still have a meaningful impact (Table 2). Furthermore, no significant differences between the two groups regarding various sociodemographic data, performance indicators, or perceived tutor behavior were found (Table 2).

As a manipulation check, most students in the intervention group reported using the interactive vector field tool (86% across all tasks) (Hahn et al., 2024), while none of the students in the control group used it. Other means of task assistance, if any, such as internet research, textbook, and lecture notes, were used equally often by both groups. Almost none of the students indicated copying answers from another student's solution, suggesting that active learning occurred in both groups.

#### 2.2.2 Data analysis

Since all data except the performance test data were given in values between 1 and 6, a linear transformation to the interval [0;1] was performed. Then, as required for parametric procedures, all scales for dependent and control variables were checked for normal distribution (see Table 1 for all scales included in the data analysis). Using the scales derived from test and scale analyses in the alpha implementation (Hahn and Klein, 2023b), all scales for analysis of alpha and beta implementation showed acceptable reliabilities (α_*C*_>0.71; Table 1). For the analysis, the vector field performance test was limited to the first 44 items, as the data for the subsequent items did not allow for meaningful interpretation. This adjustment ensured that pre- and posttest were identical and did not include any module-specific tasks. Consequently, the confidence scale was also shortened to 34 items. As do the full scales (alpha implementation; Hahn and Klein, 2023b), the abbreviated scales also show satisfactory psychometric properties.

Regarding the hypotheses, statistical analyses primarily consist of standard methods of quantitative statistics (i. e., *t*-tests) to examine the influence of group membership on the dependent variables, that means response accuracy and response confidence for pre- and posttest as well as perceived cognitive load types. Besides performance, learning gains are compared between the groups. Therefore, the absolute gain *g*, defined as the difference between pre- and post-scores, as well as Hake's gain *g*_*H*_, calculated by the quotient of absolute gain and maximum possible gain, are used (Hake, 1998). In addition, 2 × 2 analyses of variance are conducted to examine the impact of the intervention comparing pretest (*t*_0_) and posttest (*t*_1_). To gain detailed insights into group differences, covariance analyses are performed while controlling for the effects of potentially confounding variables, such as tutor behavior. Furthermore, correlations are examined to explore the relationships between variables in depth and within-subjects effects are investigated through pre- and post-comparisons of students' performance in the vector field performance test. All analysis methods described align with the proposed methods in Hahn and Klein (2023b), confirmatory analyses). All analyses are interpreted based on the guidelines provided by Cohen (1988). Pre-analyses for covariance analysis, including correlations between control and dependent variables, can be found in the Supplementary material.

## 3 Results

In the following, the data analyses are reported according to the research hypotheses H1 (Section 3.1) and H2 (Section 3.2). For H1, which concerns students' performance in vector calculus, vector field quantities, and vector field concepts, response accuracy, learning gain, and response confidence are compared between intervention and control group at pretest (*t*_0_) and posttest (*t*_1_). For H2, which addresses students' perceived cognitive load during task processing, all four types of cognitive load (ECL, ICL, GCL, and E) are compared between the groups.

### 3.1 Students' performance related to vector calculus, vector field quantities, and vector field concepts (H1)

**Figure 2:**
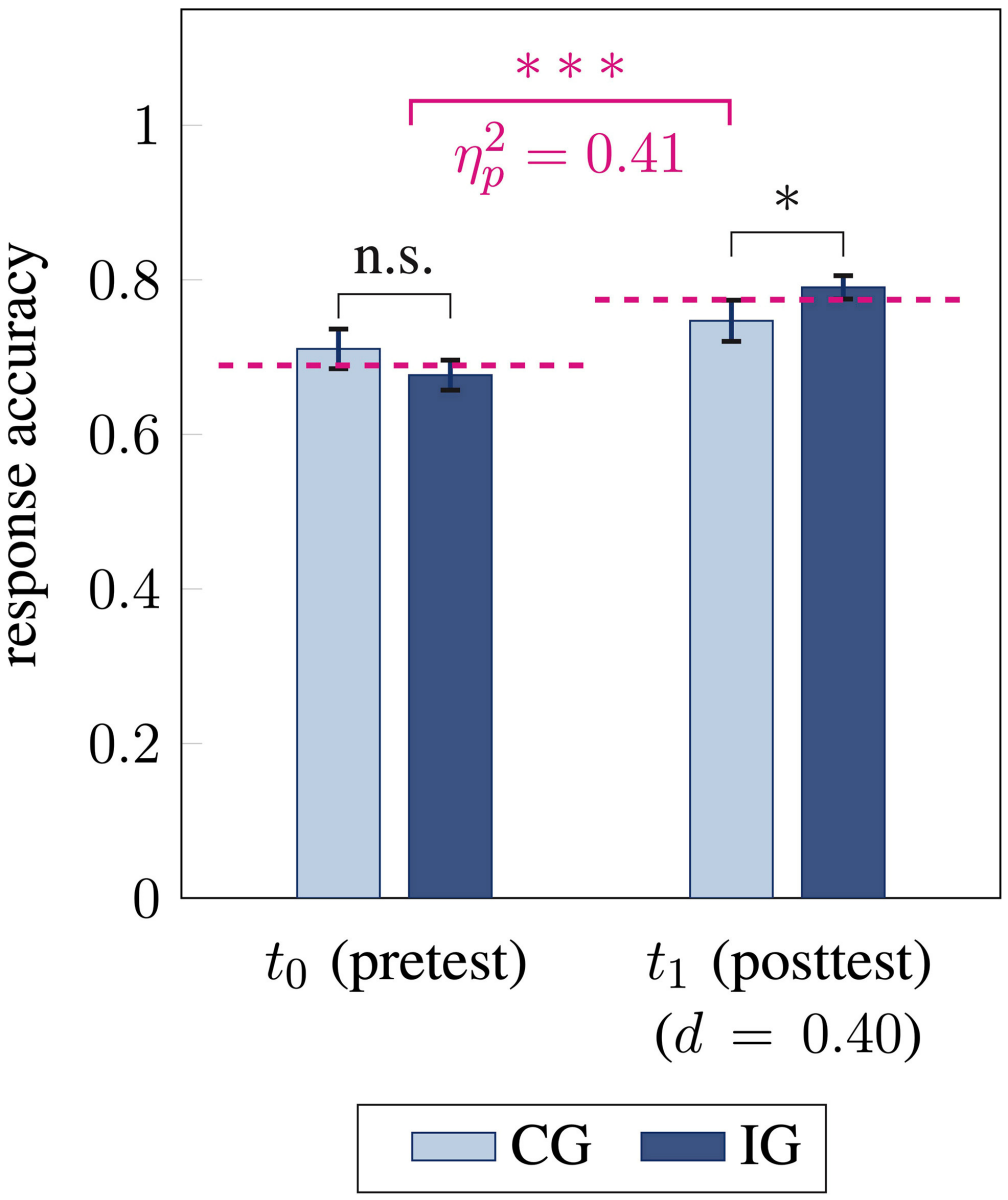
Students' response accuracy before (*t*_0_) and after (*t*_1_) the intervention for control group CG and intervention group IG. Response accuracy between the groups is compared using unpaired *t*-tests (pretest two-sided, posttest one-sided) and response accuracy between pre- and posttest (mean for *t*_0_ and *t*_1_ in violet) is compared using analyses of variance (^*^ / ^***^ / n.s. statistical significance *p* < 0.05 / *p* < 0.001 / not significant, effect sizes $\eta_{p}^{2}$ and Cohen's *d*, error bars represent 1 SEM).

After the intervention phase, students' overall response accuracy in the vector field performance test improved from 0.69 ± 0.14 to 0.77 ± 0.11, with large effect size [*F*(1, 79) = 53.72, *p* < 0.001, $\eta_{p}^{2}=0.41$; Figure 2 violet]. This achievement indicates a normalized gain *g*_*H*_ = 0.27 of small size according to Hake (1998).

**Figure 3:**
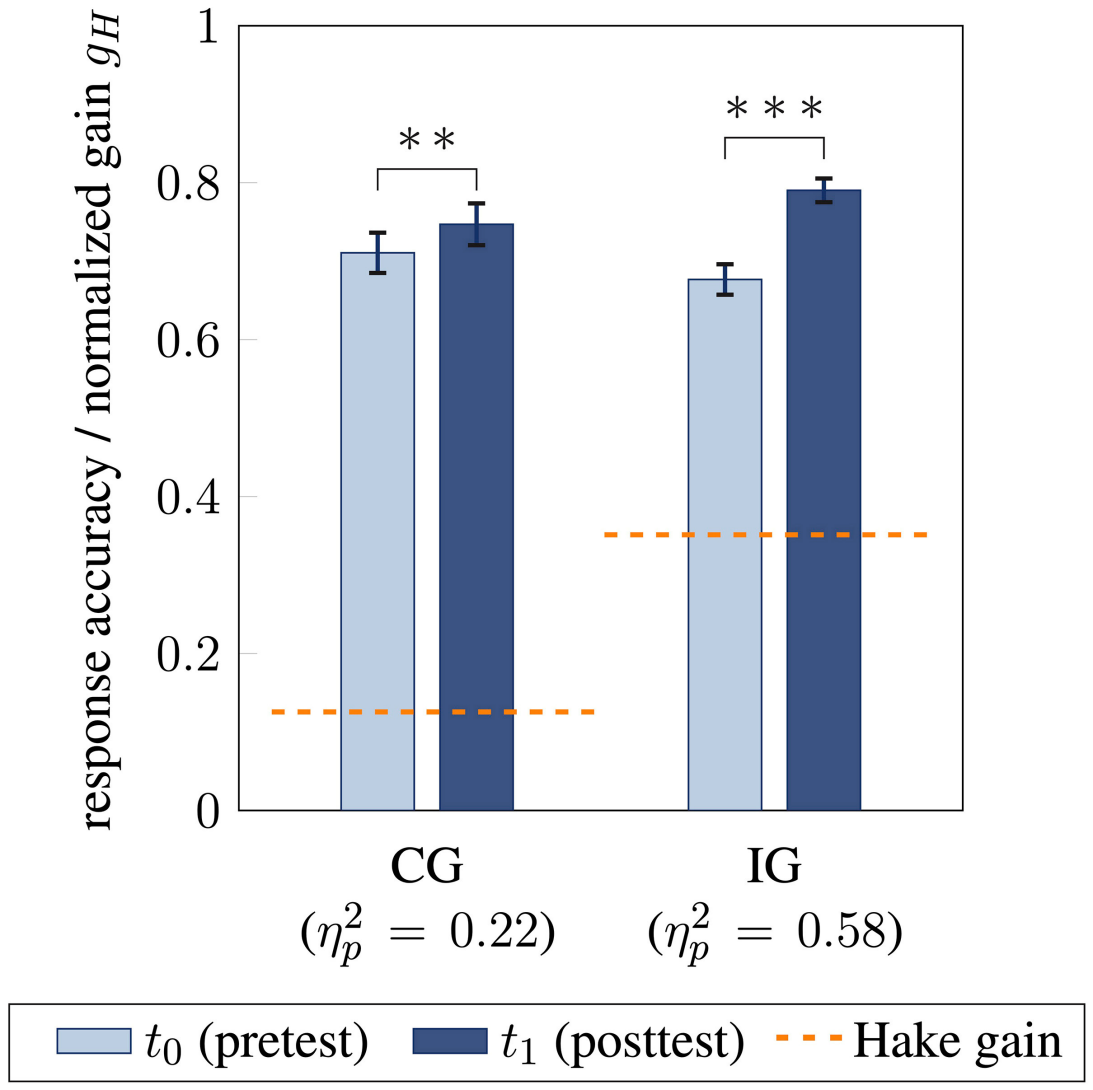
Comparison of students' response accuracy and learning gain for control group CG and intervention group IG from pre- (*t*_0_) to posttest (*t*_1_). Normalized gain *g*_*H*_ referring to Hake (1998) for both groups is visualized in orange. Response accuracy between *t*_0_ and *t*_1_ is compared using analyses of variance (^**^ / ^***^ statistical significance *p* < 0.01 / *p* < 0.001, effect size $\eta_{p}^{2}$, error bars represent 1 SEM).

When comparing intervention and control group, a large-sized interaction effect between time and group membership was found [*F*(1, 79) = 14.26, *p* < 0.001, $\eta_{p}^{2}=0.15$]. This was reflected by a larger increase in response accuracy from pre- to posttest for the intervention group [0.68 ± 0.14 to 0.79 ± 0.11; *F*(1, 50) = 68.29, *p* < 0.001, $\eta_{p}^{2}=0.58$; Cohen's *d* = 1.16] compared to the control group [0.71 ± 0.15 to 0.75 ± 0.11; *F*(1, 29) = 8.06, *p* = 0.008, $\eta_{p}^{2}=0.22$; Cohen's *d* = 0.52; Figure 3]. Referring to the interpretation of normalized gain by Hake (1998), students from the intervention group achieved a medium normalized gain of *g*_*H*_ = 0.35 (absolute gain *g* = 0.11), while students' accuracy in the control group showed a small normalized gain of *g*_*H*_ = 0.13 (absolute gain *g* = 0.04; Figure 3 orange).

After the intervention, a significant group difference regarding students' response accuracy was found, reflecting a small-sized effect [*t*(79) = 1.73, *p* = 0.04, *d* = 0.40; Figure 2]. When accounting for students' accuracy in the pretest, group differences in the posttest were further strengthened, yielding a large effect size [*F*(1, 78) = 15.86, *p* < 0.001, $\eta_{p}^{2}=0.17$], supporting the interaction effect.

**Figure 4:**
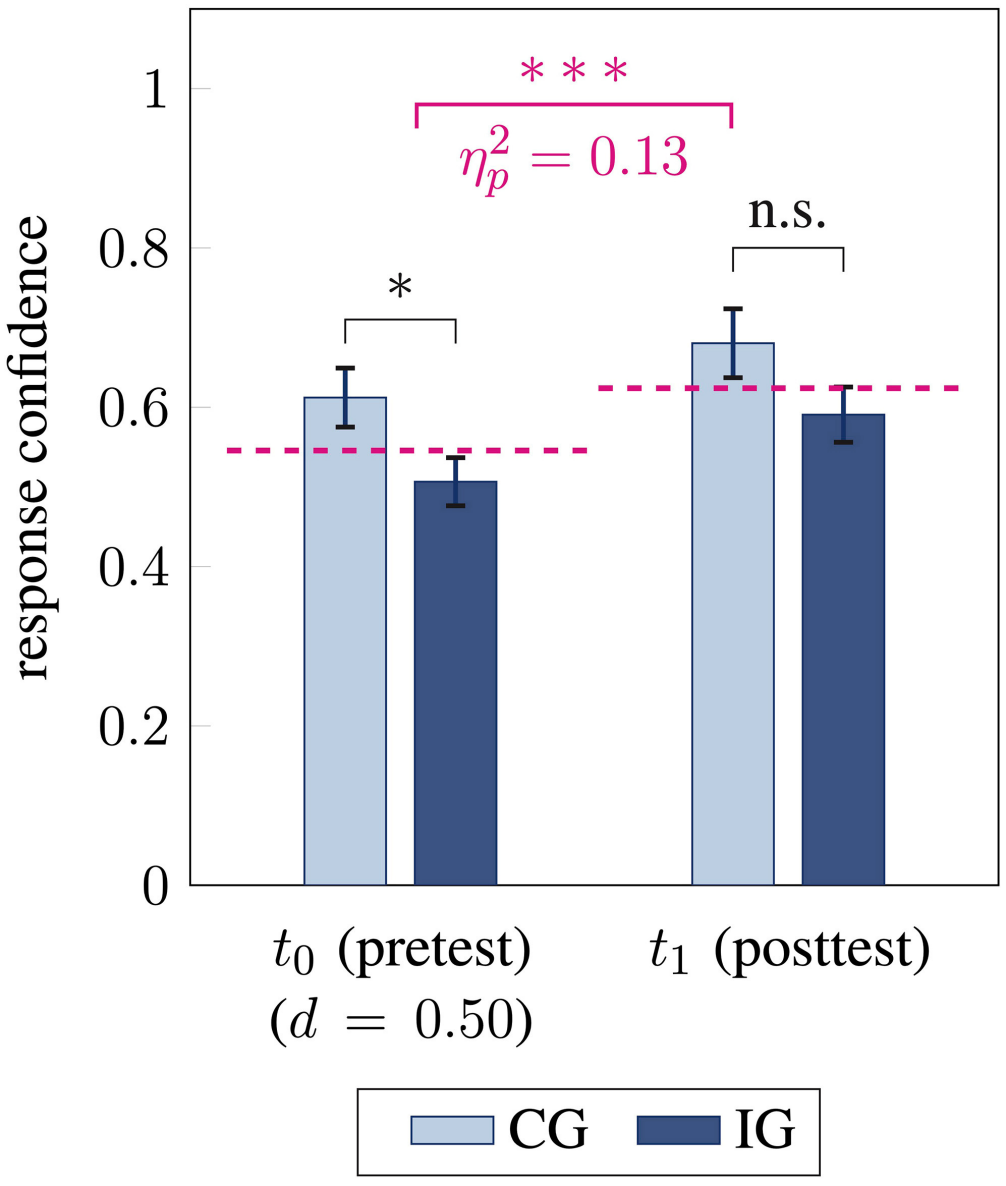
Students' response confidence before (*t*_0_) and after (*t*_1_) the intervention for control group CG and interventions group IG. Response confidence between the groups is compared using unpaired *t*-tests (pretest two-sided, posttest one-sided) and response confidence between pre- and posttest (mean for *t*_0_ and *t*_1_ in violet) is compared using analyses of variance (^*^ / ^***^ / n.s. statistical significance *p* < 0.05 / *p* < 0.001 / not significant, effect sizes $\eta_{p}^{2}$ and Cohen's *d*, error bars represent 1 SEM).

Overall, students' response confidence in the vector field performance test improved after the intervention [*F*(1, 79) = 12.24, *p* < 0.001, $\eta_{p}^{2}=0.13$], with medium effect size (Figure 4 violet). Similar large-sized effects were observed for the intervention group [*F*(1, 50) = 8.49, *p* = 0.005, $\eta_{p}^{2}=0.15$] and the control group [*F*(1, 29) = 5.76, *p* = 0.02, $\eta_{p}^{2}=0.17$]. Furthermore, no interaction effect between time and group membership for students' response confidence was found (*p* = 0.71).

Before the intervention, students in the control group were more confident about their answers in the pretest than students in the intervention group [*t*(79) = −2.18, *p* = 0.03, *d* = 0.50; Figure 4]. After the intervention, the group difference in students' response confidence diminished (*p* = 0.06). When students' accuracy in the pretest was taken into account, the group difference in response confidence further reduced (*p* = 0.19).

In both the pre- and the posttest, students' response accuracy was moderately correlated with their response confidence (pre *r* = 0.44, *p* < 0.001; post *r* = 0.38, *p* < 0.001). This correlation was large- and medium-sized for the intervention group (pre *r* = 0.52, *p* < 0.001; post *r* = 0.38, *p* = 0.006) and small- and large-sized for the control group (pre *r* = 0.26, *p* = 0.16; post *r* = 0.51, *p* = 0.004)

### 3.2 Students' perceived cognitive load during task processing (H2)

**Figure 5:**
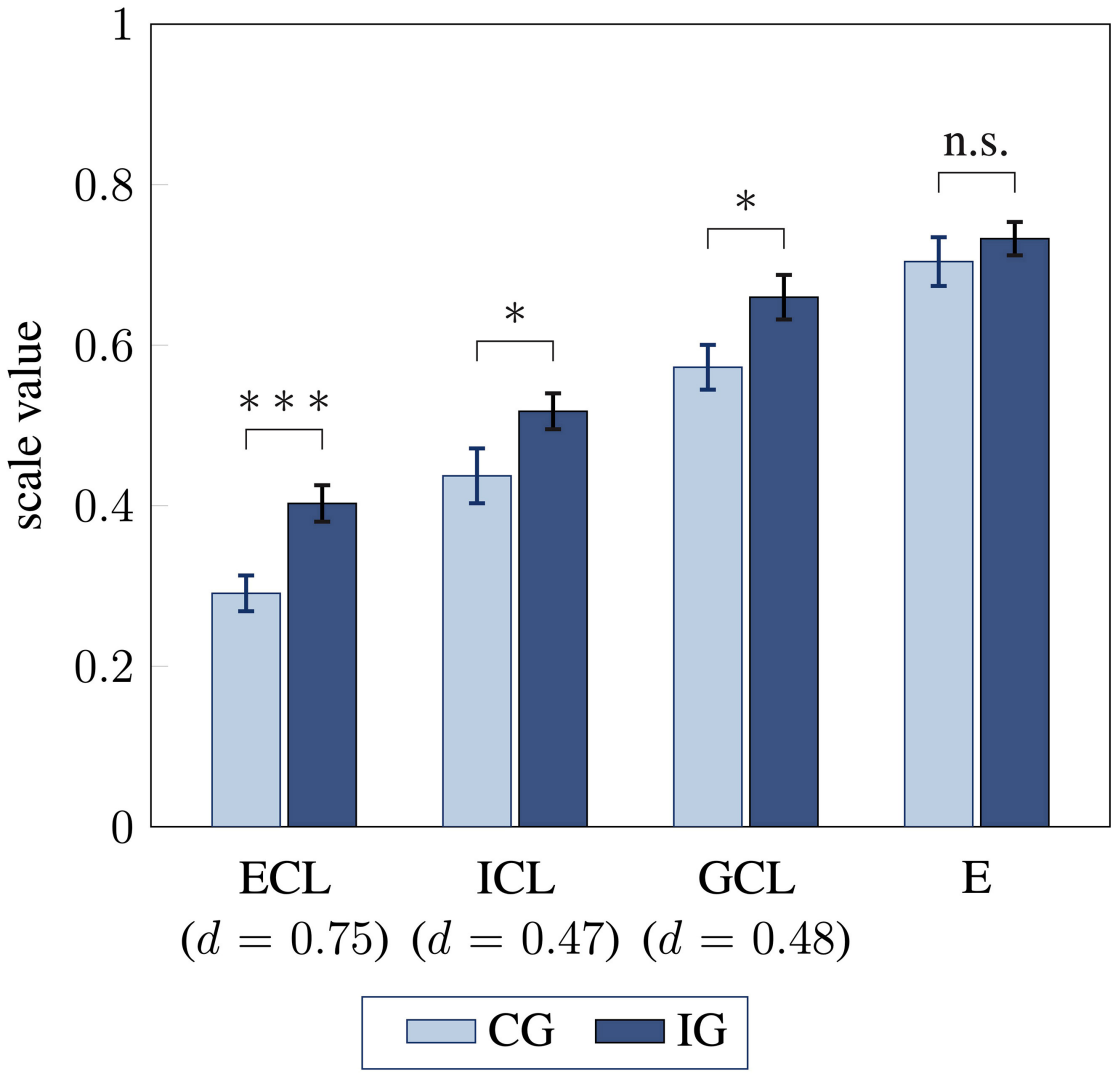
Students' perceived cognitive load for control group CG and intervention group IG. Extraneous cognitive load (ECL), instrinsic cognitive load (ICL), germane cognitive load (GCL), and cognitive effort (E) are compared between the groups using unpaired *t*-tests (one-sided; ^*^ / ^***^ / n.s. statistical significance *p* < 0.05 / *p* < 0.001 / not significant, effect size Cohen's *d*, error bars represent 1 SEM).

Students in the intervention group reported significantly higher extraneous, intrinsic, and germane cognitive load compared to the control group (Figure 5). Group comparison of extraneous cognitive load showed a medium-sized effect [*t*(79) = 3.27, *p* < 0.001, *d* = 0.75]. Both intrinsic and germane load differed between the groups with small effect sizes [*t*(79) = 2.05, *p* = 0.02, *d* = 0.47 and *t*(79) = 2.08, *p* = 0.02, *d* = 0.48, respectively]. However, when accounting for response accuracy in the pretest, a plausible predictor of performance, no significant group differences in intrinsic cognitive load were found (*p* = 0.07). Moreover, when tutor effects were considered, significant group differences in germane cognitive load were further strengthened, showing a medium-sized effect [*F*(1, 70) = 8.71, *p* = 0.004, $\eta_{p}^{2}=0.11$]. Additionally, germane cognitive load was overall positively correlated with students' individual absolute learning gain (*r* = 0.25, *p* = 0.02), but no correlation with posttest response accuracy was found (*p* = 0.97). Students' effort expended during task completion did not differ between intervention and control group (*p* = 0.21), with both groups showing high values above 0.70.

## 4 Discussion

### 4.1 Impact of multi-representational learning tasks on students' performance (H1)

Before the intervention, students demonstrated high prior knowledge of visual representations of vector fields and vector decomposition (Table 2). However, their ability to evaluate the curl of a vector field diagram was limited, with only 31% accuracy. This aligns with findings in previous research, which also highlighted challenges in students' ability to interpret vector field diagrams (Baily et al., 2016; Hahn and Klein, 2023a; Klein et al., 2018, 2019; Singh and Maries, 2013). After the intervention, both overall response accuracy and confidence in vector calculus concepts significantly improved, with large and medium effect size, respectively. These results underline the value of instructional support for vector calculus in physics study entry phase, consistent with conclusions from prior studies (Bollen et al., 2018; Dray and Manogue, 2023; Hernandez et al., 2023; Singh and Maries, 2013).

When comparing intervention and control group, students who worked with multi-representational learning tasks, including sketching activities and an interactive vector field simulation, achieved significantly higher scores after the intervention and showed a higher normalized learning gain. With a value of 0.35, the gain of the multi-representational learning tasks corresponds to the normalized gain of interactive engagement methods in previous physics education studies (e.g., Coletta et al., 2007; Hake, 1998; Hernández et al., 2021; Núñez et al., 2021; Sahin, 2010). In contrast, the control group, which worked with traditional calculations-based learning tasks, showed a low normalized gain of 0.13, even falling below the results from traditional courses in earlier studies (Coletta et al., 2007). These findings highlight the value of instructional support through multiple representations, sketching activities, and interactive visualizations in promoting learning of vector calculus and other complex physics concepts. Using Cohen's *d* instead of Hake's gain further supports the abovementioned conclusions (Nissen et al., 2018). Moreover, a large-sized interaction effect between time and group membership further emphasized the impact of multi-representational tasks on students' learning. These findings support hypothesis H1 and align with theoretical frameworks from cognitive psychology, which advocate for combining multiple representations, sketching activities, and interactive visualizations as effective learning approaches to enhance conceptual understanding and representational competencies of abstract concepts (e.g., Ainsworth, 1999; Ainsworth and Scheiter, 2021; Kohnle et al., 2020; Stieff, 2011). Therefore, multiple representations in task-based learning are particularly beneficial for introductory physics students, as they address common challenges students face when learning about these complex concepts (e.g., Bollen et al., 2015; Pepper et al., 2012; Singh and Maries, 2013).

In previous studies, students achieved impressive scores in evaluating divergence, answering conceptual questions, and completing transfer tasks after multi-representational, sketching-based instruction (e.g., 88% on divergence evaluation, 85% on conceptual questions, and 81% on partial derivatives tasks; Hahn and Klein, 2023a). The results from this study, with a score of 79% on the vector calculus performance test, demonstrate comparable effectiveness of the multi-representational learning tasks in a typical physics university lecture setting. These findings offer initial evidence for the transfer of results from previous clinical studies on vector fields (Hahn and Klein, 2023a; Klein et al., 2018, 2019) to regular university teaching contexts. Additionally, the successful extension of previous divergence-focused instructions to other vector calculus concepts can be inferred. However, comparisons between this study and that of Hahn and Klein (2023a) are limited due to differences in study conditions. This applies in particular to the sample (first-year students vs. second-year students in this study). Furthermore, performance assessment in the study by Hahn and Klein (2023a) took place immediately after the intervention, i.e., a maximum of 60 min elapsed between the pretest and the posttest. In this study, in contrast, the posttest was conducted 1 week after the fourth learning task was discussed in the recitation, i.e., 6 weeks passed between the pre- and the posttest. The latter indicates that the learning outcomes measured in this study likely reflect sustainable, long-term learning effects rather than short-term gains. Moreover, it should be emphasized that this effect was demonstrated in a much more realistic setting compared to the study by Hahn and Klein (2023a), as the students worked on the tasks independently without external control.

Compared to previous studies (Hahn and Klein, 2023a; Klein et al., 2018, 2019), the multi-representational learning tasks in this study were enhanced by an interactive vector field visualization tool. Therefore, this study provides initial indications of the educational value of the tool. The findings here align with those from Hahn et al. (2024), indicating a good alignment of the simulation, the learning tasks, and students' prior knowledge. Additionally, students' indication of high perceived educational impact of the visualization tool can initially be confirmed with performance measures.

Group comparisons in the vector field performance test revealed that multi-representational learning tasks led to higher learning gains and performance scores. However, students in the intervention group did not show greater confidence in their answers compared to the control group. In contrast, students who worked with traditional tasks reported higher response confidence both before and after the intervention. Notably, these group differences diminished after the intervention, suggesting a greater gain in metacognitive abilities among students in the intervention group. Additionally, significant positive correlations between accuracy and confidence were found before and after the intervention for both groups, mirroring results from previous studies (Klein et al., 2017, 2019; Lindsey and Nagel, 2015). This suggests that students were generally aware of their performance, i. e., those who answered correctly displayed high response confidence and those with incorrect answers had lower confidence. Such high metacognitive abilities are a key outcome of effective learning, as they enable learners to regulate and improve their learning processes (Lindsey and Nagel, 2015; May and Etkina, 2002).

### 4.2 Impact of multi-representational learning tasks on perceived cognitive load during task processing (H2)

In the recitations, a short questionnaire on perceived cognitive load was administered before task discussion. Students who worked with multi-representational learning tasks and those working with traditional learning tasks reported similar levels of effort invested during task completion. Values above 0.70 suggest that considerable amount of cognitive resources were allocated to meet the task demands (Paas and Van Merriënboer, 1994). As mental effort is influenced by prior knowledge and experiences regarding the tasks' requirements (Paas and Van Merriënboer, 1994), these results imply a high level of alignment between the learning tasks and learners' prior knowledge. Moreover, as indicated by high, but not excessive, values, the tasks encouraged learners to exert cognitive effort, which is crucial for the construction of cognitive schemata and, consequently, for learning (Sweller, 2010).

When comparing intervention and control group, students who engaged with multi-representational learning tasks, including sketching activities and an interactive vector field tool, reported higher levels of extraneous, intrinsic, and germane cognitive load during task processing. The group difference in germane cognitive load suggests that students working with multi-representational learning tasks were able to allocate more working memory resources to processing the subject matter (Sweller, 2010). This conclusion is further supported when considering the tutor behavior. Particularly, this result aligns with the abovementioned findings that students who worked with multi-representational learning tasks showed higher learning achievement compared to the control group and aligns with results from previous studies in the context of vector fields (Hahn and Klein, 2023a). Following theories from cognitive psychology (e.g., Ainsworth, 1999; Ainsworth and Scheiter, 2021; Stieff, 2011), these results further underline the value of instructional support through multiple representations, sketching activities, and interactive visualizations for complex and field-related concepts. Additionally, the positive correlation between germane cognitive load and individual absolute learning gain for both groups indicates that students were deeply engaged in metacognitive processes (Leppink, 2017). This suggests that, beyond simply gaining knowledge, students were also able to correspondingly estimate their improvement in understanding, consistent with findings from previous studies (e.g., Huang et al., 2013). These results further support the conclusion made above that students in both the intervention and the control group demonstrated high metacognitive abilities.

Following Cognitive Load Theory (Sweller, 2010), elevated values in extraneous cognitive load suggest that the design of multi-representational learning tasks imposed greater cognitive demands during task processing compared to traditional, calculation-based tasks. However, despite increased extraneous cognitive load, students in the intervention group reported an amplified perceived learning impact (germane cognitive load) and achieved higher learning gains. These results do not align with findings from previous studies in the context of vector fields (Hahn and Klein, 2023a). However, similar findings have been observed in previous studies where realistic graphics and immersive learning environments, which induced task-irrelevant cognitive load, led to improved performance (Makransky et al., 2019; Skulmowski and Rey, 2020a). Recent research on disfluency suggests that under certain circumstances harder-to-perceive learning materials are able to trigger learners to invest more cognitive effort, ultimately improving learning outcomes (Skulmowski and Rey, 2018). Specifically, interactive digital learning tools were found to promote such an effect (Skulmowski and Xu, 2022). As the interactive vector field visualization tool was used in 86% of all task completions, this may also apply to this study. These findings align with recent research in educational psychology advocating for the differentiation of extraneous cognitive load components, particularly in digitally-supported learning environments (Skulmowski and Rey, 2020b; Skulmowski and Xu, 2022). This approach might be particularly promising for learning environments such as the one used here, i. e. learning tasks combining digital and text-based learning, as well as incorporating various methods. Additionally, high values of extraneous cognitive load might reflect unfamiliarity with the instructional format, that means text- and representation-rich tasks that require qualitative reasoning and sketching (Orru and Longo, 2019). Since verbalization plays a crucial role in physics and mathematics reasoning (Sirnoorkar et al., 2020), this interpretation suggests the need for targeted support, such as an introduction to the task format. However, further empirical research is required to clarify this line of reasoning. Regardless of the group comparison, extraneous load values below 0.40 can generally be classified as low—for students engaged in lab work or smartphone-based experimental exercises similar or higher values have been reported (Kaps and Stallmach, 2022; Thees et al., 2020).

Students who worked with multi-representational learning tasks perceived higher intrinsic cognitive load, but this group difference was reduced when taking students' response accuracy in the pretest into account. That means, when baseline differences in students' prior knowledge were adjusted, the perceived complexity of the learning subject did not differ significantly between students who worked with multi-representational and traditional learning tasks. However, these findings contradict hypothesis H2 and suggest that sketching activities were not able to unfold their expected relieving effect, as emphasized in theoretical considerations (Bilda and Gero, 2005) and found in previous studies in the context of vector fields (Hahn and Klein, 2023a). The perceived complexity of the learning subject, however, also depends on what the learner associates with the learning subject. With higher prior knowledge—gained through working on multi-representational learning tasks—students might consider additional aspects as part of the subject matter, aspects that students working on the calculation-based tasks might disregard. Such aspects, for example, qualitative evaluation of vector field diagrams, may add complexity to the learning subject, resulting in a higher perceived intrinsic cognitive load (Endres et al., 2023).

### 4.3 Conclusion and future work

In this work, the impact of multi-representational learning tasks on vector calculus implemented in weekly recitations of an electrodynamics course in introductory physics studies was investigated. Specifically, multi-representational learning was integrated with sketching activities and an interactive vector field visualization tool. Analyses focused on students' response accuracy and confidence in a vector calculus performance test, and students' perceived cognitive load during task processing.

Besides showing an overall positive impact of the intervention on students' achievement, multi-representational learning tasks led to significantly higher learning outcomes and promoted amplified learning gains. Further, students who worked with these learning tasks perceived higher germane cognitive load, reflecting that they devoted more working memory resources to the subject to be learned, despite perceiving higher intrinsic and extraneous cognitive load. These results support a nuanced perspective on the relationship between the three types of cognitive load, suggesting that certain aspects of extraneous load–such as those induced by interactive visualizations–can facilitate deeper processing and, consequently, foster learning. As such, the findings provide valuable insights for further research in educational psychology, particularly in exploring the nuanced interplay between different components of cognitive load and their effects on learning outcomes. Future studies should explore in greater depth how instructional design strategies, such as multiple representations and and interactive visualizations in task-based learning, shape learners' cognitive and metacognitive processes, and examine the role of task format familiarity in mitigating extraneous cognitive load.

The primary limitation of the study lies in its field study nature, as it was implemented within university physics curricula. While students indicated task assistance, the exact learning process remained unknown and nontransparent. As a result, the findings lack internal validity, meaning that causal interpretations of the results are not unambiguous. Consequently, future studies should include analyses of learning outcomes across different universities with varying curricula to elaborate the conclusions and implications for instructors made here.

Concerning the value of this article for research on learning with multiple representations, it extends previous studies (Bollen et al., 2018; Hahn and Klein, 2023a; Klein et al., 2018, 2019) investigating multi-representational instructions by providing empirical evidence regarding the effectiveness of such an approach in university teaching, compared to a traditional control group. Specifically, multi-representational learning tasks, including sketching activities and interactive visualizations, were shown to enhance students' performance in tasks addressing conceptual understanding and representational competencies related to abstract physics concepts. At this point, it should be emphasized that the effectiveness of the learning tasks was demonstrated in a very realistic setting. In contrast to previous clinical studies (Hahn and Klein, 2023a; Klein et al., 2018, 2019), there was no control over learning time; the tasks were completed at students' own responsibility and without supervision. Although the learning tasks used here in this study required prior knowledge about vector field representations, thus targeting university science students, some implications can also be extended beyond physics teaching. For instructors, learning tasks incorporating multiple representations, sketching activities, and interactive visualization tools are highly recommended. Particularly, for teaching complex concepts that are typically calculation-based or not visually introduced, multi-representational, sketching- and simulation-based learning proves to be a promising method that can also be applied outside university settings, such as in school education. When using this approach, it is recommended to closely link sketching tasks with interactive simulations, such as creating a drawing from the tool (Kohnle et al., 2020).

For STEM education, such learning tasks could provide meaningful support in undergraduate physics lectures, as vector calculus is fundamental to numerous fields of physics, for example, electrodynamics and fluid mechanics. However, empirical research on the application of vector calculus concepts in electrodynamics or other physics fields after completing multi-representational learning tasks is still lacking. Addressing this gap should be a priority for future studies.

## 5 Preregistration and deviations from the original analysis plan

This study was preregistered, meaning that the research questions, methodology, and study materials were reviewed and approved before data collection began (Hahn and Klein, 2023b). The preregistration process ensures transparency and reliability by committing to a specific research design in advance. Accordingly, the study was conducted as outlined in the preregistration, using all specified test instruments and analysis procedures.

However, one deviation from the preregistered plan became necessary due to a high dropout rate. The core sample—comprising students who completed all performance tests and learning tasks—was reduced to only 10 participants (*N*_IG_ = 6, *N*_CG_ = 4). This sample size did not meet fundamental statistical assumptions, making some of the planned analyses infeasible. As a result, contrary to the preregistered plan, analyses were restricted to intervention phase I. This means that the originally planned learning gain analyses for intervention phase II and comparisons between both intervention phases could not be conducted. Importantly, this adjustment does not alter the validity of the study's core findings, as the guiding question and research hypotheses could still be addressed with the available data.

Beyond this change in the scope of analyses, the methodology and study implementation remained fully aligned with the preregistration. All test and scale analyses specified in the preregistration were conducted as planned. The cognitive load scales demonstrated acceptable reliabilities (Cronbach's α_*C*_>0.71). However, due to a higher frequency of missing values in the later items of the vector field performance test, analyses were limited to the first 44 items. Similarly, the confidence scale was shortened to 34 items to ensure meaningful interpretation. These adjustments were necessary to maintain the validity of the analysis, and critically, the shortened scales exhibited psychometric properties comparable to the full versions.

Despite these modifications, all analyses were conducted within the preregistered confirmatory framework, and the study's methodological rigor remains intact.

## Data Availability

The raw data supporting the conclusions of this article will be made available by the authors, without undue reservation.
