# Supplementary material for: The impact of multiple representations on students' understanding of vector field concepts: Implementation of simulations and sketching activities into lecture-based recitations in undergraduate physics
Source: Front Psychol. 2025 Apr 25;16:1544764. doi: 10.3389/fpsyg.2025.1544764 (PMC12061973; doi:10.3389/fpsyg.2025.1544764)
Supplement: Supplementary file 1 [file Data_Sheet_1.pdf]

Supplementary Material

The Supplementary material follows the structure given below. First, relevant correlations as pre analyses for covariance analysis are given. Then, multi-representational and traditional calculation-based learning tasks are presented in English (translated from originally German).

1 Pre analyses for covariance analysis: correlations

2 Study material

2.1 Multi-representational learning tasks

2.2 Traditional learning tasks

1 PRE ANALYSES FOR COVARIANCE ANALYSIS: CORRELATIONS

Table S1. Correlations between control variables and dependent variables.

	Dependent variable				
Control variable	V_1	C_1	ECL	ICL	GCL
Mean age	-.22	-.13	-.04	-.01	.12
No. of semesters studied	-.13	.14	-.07	.15	.18
Average grade for university entrance	-.20	-.22	.02	-.08	.06
Tutor behavior	-.21	.16	-.08	.23	.26*
V_0	.74***	.33**	-.20	-.27*	-.17
C_0	.36***,[a]	.68***,[a]	-.05	-.04	-.29**,[b]

* / ** / *** significant correlation $p < 0.05$ / $p < 0.01$ / $p < 0.001$

[a]excluded from covariance analyses due to a violation of group independence

[b]excluded from covariance analyses due to a violation of the homogeneity of the regression slopes

2 STUDY MATERIAL

All learning tasks are translated from originally German. The study was conducted in German.

2.1 Multi-representational learning tasks

Learning task: Divergence of a vector field
In this exercise, you will study the divergence of vector fields using a simulation (`https://wwwuser.gwdguser.de/physik.didaktik/oer/v3ctor/`). You *may* use the simulation for each subtask. For some tasks you *have to* use the simulation. In that case, it will be explicitly stated.

(a) Familiarize yourself with the simulation. To do so, enter various vector fields and try out all the features. The information icons can give you some orientation.
Tip: Start with a vector field with an x-component $A_x = 1$ and a y-component $A_y = 0$ and move on to other, non-constant vector fields afterwards.

(1) Comment on the following statement: "The space in between two vectors is empty."

(2) Draw a two-dimensional vector field $\vec{A}_1(x, y)$ consisting of only an x-component which in turn depends on the y-coordinate. Give $\vec{A}_1(x, y)$ in the form of $\vec{A}_1(x, y) = A_{1,x}\hat{e}_x + A_{1,y}\hat{e}_y$. Next, draw a two-dimensional vector field $\vec{A}_2(r, \varphi)$ consisting of only a φ-component dependent on the r-coordinate and give it in the form of $\vec{A}_2(r, \varphi) = A_{2,r}\hat{e}_r + A_{2,\varphi}\hat{e}_\varphi$.

(b) In Cartesian coordinates, the divergence of a vector field ($\operatorname{div}\vec{A} = \vec{\nabla} \cdot \vec{A}$) at the location (x, y) is defined as the sum of the partial derivatives

$$\operatorname{div}\vec{A}(x, y) = \frac{\partial}{\partial x}A_x + \frac{\partial}{\partial y}A_y\,.$$

Consider the vector field $\vec{B}(x, y)$ with

$$\vec{B}(x, y) = -k\,(x\hat{e}_x + y\hat{e}_y)\,, \qquad \text{where } k \in \mathbb{R} \text{ is constant}\,.$$

(1) Draw $\vec{B}(x, y)$ for $k = -1$.

(2) Choose any point in your sketch and draw the field components for the vector at this location as well as for the vectors in its immediate vicinity.

(3) Using your sketch, give a qualitative assessment of how the field components change along the coordinate directions, i.e. indicate whether the partial derivatives $\frac{\partial}{\partial x}B_x$ and $\frac{\partial}{\partial y}B_y$ are positive, negative or zero. Use this result to determine whether the divergence at your chosen location is positive, negative or zero. Verify your result using the simulation and a calculation.

(c) Now look at the vector field $\vec{C}(x, y)$, where

$$\vec{C}(x, y) = xy\hat{e}_x + \hat{e}_y$$

in the simulation.

For each of the four quadrants, assess how the field components change in the directions of x and y and conclude whether the divergence within the quadrants is positive, negative or zero. Use a calculation to verify and justify your result.

(d) Inside the pipe geometry shown, there is a stationary, i.e. time-independent, laminar flow in the positive x-direction, which is described by a velocity vector field $\vec{v}(x, y)$ with $v_y = 0$ in the straight sections I and III of the pipe.

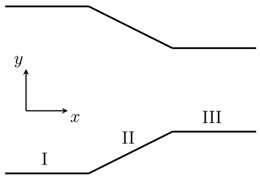

(1) Draw the field $\vec{v}(x, y)$, where $|\vec{v}_{\mathrm{III}}| > |\vec{v}_{\mathrm{I}}|$.

(2) For flows of this type, the continuity equation applies: $\operatorname{div}\vec{v}(x, y) = 0$. Explain how the divergence of the field can be zero even though the horizontal velocity component increases from I to III.

(e) In polar coordinates, the divergence of a vector field $\vec{A}(r, \varphi)$ at a location (r, φ) is defined as follows:

$$\operatorname{div}\vec{A}(r, \varphi) = \frac{1}{r}\frac{\partial}{\partial r}(rA_r) + \frac{1}{r}\frac{\partial}{\partial \varphi}A_\varphi\,.$$

The above vector field $\vec{B}$ is given in polar coordinates by $\vec{B}(r, \varphi) = -kr\hat{e}_r$ (where $k = -1$). In subtask (b) you studied the relationship between the divergence and the change of the field components in the Cartesian coordinate directions. Using a sketch of $\vec{B}(r, \varphi)$, describe how you would graphically interpret the definition of divergence in polar coordinates. Also consider the characteristics of the definition in polar coordinates in your explanation. Calculate $\operatorname{div}\vec{B}(r, \varphi)$ and compare the result to your result from subtask (b).

Learning task: Gauss' theorem
In this exercise, you will study Gauss' theorem. For this, you will use the vector field simulation from exercise sheet 1 (`https://wwwuser.gwdguser.de/physik.didaktik/oer/v3ctor/`). You *may* use the simulation for each subtask. For some tasks you *have to* use the simulation. In that case, it will be explicitly stated.

Consider the vector field $\vec{F}(x,y)$, where

$$\vec{F}(x,y) = x\hat{e}_x + 2\hat{e}_y\,.$$

(a) The flux Φ of a two-dimensional vector field $\vec{B}(x,y)$ trough the boundary curve $C = \partial A$ of an area A is calculated using the surface integral

$$\Phi = \int_{\partial A} \vec{B}(x,y) \cdot d\vec{n}\,,$$

where $d\vec{n} = \hat{n}dl$, and the normal vector of the curve $\hat{n}$ is directed outwards.

(1) Draw the field $\vec{F}(x,y)$.

(2) Add a rectangle of your choice to your sketch and draw the normal vectors of the curve as well as the projection of the field components onto these normal vectors to each of the four sides of the rectangle.

(3) Determine the inflows and outflows of the field components at the edges of the rectangle: Does the rectangle experience an overall inflow or outflow?

(b) In two dimensions, Gauss' theorem describes the relationship between the divergence of a vector field $\vec{B}(x,y)$ within an area A and the flux of the vector field through the (closed) boundary curve of the area ∂A,

$$\int_A \operatorname{div} \vec{B}\, dA = \int_{\partial A} \vec{B} \cdot d\vec{n}\,.$$

(1) Give a comprehensible explanation of $\int_A \operatorname{div} \vec{B}\, dA$. To do this, use the qualitative interpretation of divergence from exercise sheet 1 and of surface integrals.

(2) Using $\vec{F}(x,y)$ as an example, explain the relationship between the change in field components and the flux through an area. Draw a sketch.

(3) Use the simulation to determine the flux of $\vec{F}(x,y)$ through the boundary of a square with an edge length of 1. Use a calculation to show that the result is equal to the left side of Gauss' theorem.

(c) Gauss' theorem in three dimensions is defined as

$$\int_V \operatorname{div} \vec{B}\, dV = \int_{\partial V} \vec{B} \cdot d\vec{n}$$

for a volume V with a closed surface ∂V. From this, we get the coordinate-independent form of the divergence

$$\operatorname{div} \vec{B} = \lim_{V \to 0} \frac{1}{V} \int_{\partial V} \vec{B} \cdot d\vec{n}\,.$$

(1) In subtask (b) you studied the relationship between the change in field components and the flux through an area using Gauss' theorem. Explain how this interpretation of Gauss' theorem can be applied in three dimensions.

(2) Use the limit of the function above to explain why the divergence of a field can be described as a measure of outgoingness at a certain location.

(d) The velocity of the water stream shown on the right is given by the depicted velocity vector field $\vec{v}(x,y)$.

(1) The continuity equation $\operatorname{div} \vec{v}(x,y) = 0$ applies to the water stream, and therefore $\int_{\partial A} \vec{v} \cdot d\vec{n} = 0$. Explain how the flux through the rectangle A can be zero even though there is an overall outflow along the horizontal edges.

(2) Is $\int_{\partial A} \vec{v} \cdot d\vec{n}$ still zero when (i) A is very small, (ii) A is circular, and (iii) A is partially located outside the confined water stream? Give reasons for your answer.

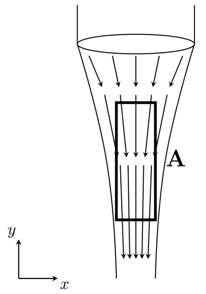

Learning task: Curl of vector fields
In this exercise, you will study the curl of vector fields. For this, you will use the vector field simulation from exercise sheets 1 and 2 (`https://wwwuser.gwdguser.de/physik.didaktik/oer/v3ctor/`). You *may* use the simulation for each subtask. For some tasks you *have to* use the simulation. In that case, it will be explicitly stated.

In Cartesian coordinates, the curl of a vector field ($\mathrm{curl}\,\vec{A} = \vec{\nabla} \times \vec{A}$) at the location (x, y, z) is defined by component-wise subtraction of the partial derivatives:

$$\mathrm{curl}\,\vec{A}(x,y,z) = \left(\frac{\partial A_z}{\partial y} - \frac{\partial A_y}{\partial z}\right)\hat{e}_x + \left(\frac{\partial A_x}{\partial z} - \frac{\partial A_z}{\partial x}\right)\hat{e}_y + \left(\frac{\partial A_y}{\partial x} - \frac{\partial A_x}{\partial y}\right)\hat{e}_z\,.$$

(a) Give a simplified definition of the curl $\mathrm{curl}\,\vec{A}$ of a vector field $\vec{A}(x, y, z = 0)$ in two dimensions.

(b) Consider the vector field $\vec{B}(x, y, z)$, where

$$\vec{B}(x,y,z) = -y\hat{e}_x + x\hat{e}_y\,.$$

(1) Draw $\vec{B}(x, y, z)$ in the x-y-plane.

(2) Add a paddle wheel (as seen on the right) to any location within the field and draw the field components to the vectors in the vicinity of the wheel. Imagine that the vector field interacts with the wheel like a fluid. Indicate the field components that act on the paddle wheel. Does the paddle wheel spin? Give reasons for your answer!

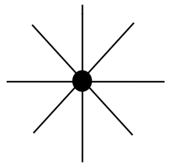

(3) Using your sketch, give a qualitative assessment of how the field components change in the coordinate directions, i.e. indicate whether the partial derivatives $\frac{\partial B_x}{\partial y}$ and $\frac{\partial B_y}{\partial x}$ are positive, negative or zero. Use this result to determine whether the curl at your chosen location is positive, negative or zero. Verify your result using the simulation and a calculation.

(4) Is there a location within the vector field $\vec{B}(x, y, z)$, where the curl is zero? Give reasons for your answer!

(c) Now look at the vector field $\vec{C}(x, y, z = 0)$, where

$$\vec{C}(x,y,z=0) = xy\hat{e}_x + \hat{e}_y.$$

in the simulation.

For each of the four quadrants, assess how the field components change in the directions of x and y and conclude whether the curl within the quadrants is zero, points in the positive direction of $\hat{e}_z$, or points in the negative direction of $\hat{e}_z$. Use a calculation to verify and justify your result.

(d) Consider a stationary, i.e. time-independent, laminar air flow which and its velocity vector field $\vec{v}(x, y, z = 0)$ that inertially only has a horizontal velocity different from zero. A disturbance deflects the flow in the negative y direction while the horizontal velocity does not change.

(1) Draw $\vec{v}(x, y, z = 0)$ in the x-y-plane.

(2) Explain why the disturbance causes the formation of vortices.

(e) In cylindrical coordinates, the curl of a vector field $\vec{A}(r, \varphi, z = 0)$ at a location $(r, \varphi, z = 0)$ is defined as

$$\mathrm{curl}\,\vec{A}(r,\varphi,z=0) = \frac{1}{r}\left(\frac{\partial}{\partial r}(rA_\varphi) - \frac{\partial A_r}{\partial \varphi}\right)\hat{e}_z\,.$$

The above vector field $\vec{B}$ is given in cylindrical coordinates by $\vec{B}(r, \varphi, z = 0) = r\hat{e}_\varphi$. In subtask (b) you studied the relationship between the curl and the change in the field components in the coordinate directions. Using a sketch of $\vec{B}(r, \varphi, z = 0)$, describe how you would graphically interpret the definition of curl in (two-dimensional) cylindrical coordinates. Also consider the characteristics of the definition in cylindrical coordinates in your explanation. Calculate $\mathrm{curl}\,\vec{B}(r, \varphi, z = 0)$ and compare the result to your result from subtask (b).

Learning task: Stokes' theorem

In this exercise, you will study Stokes' theorem. For this, you will use the vector field simulation from exercise sheets 1, 2, and 3 (`https://wwwuser.gwdguser.de/physik.didaktik/oer/v3ctor/`). You *may* use the simulation for each subtask. For some tasks you *have to* use the simulation. In that case, it will be explicitly stated.

Consider the vector field $\vec{F}(x,y,z)$, where

$$\vec{F}(x,y,z) = y\hat{e}_x + 2\hat{e}_y\,.$$

(a) The circulation Ψ of a three-dimensional vector field $\vec{B}(x,y,z)$ along a closed boundary curve $C = \partial A$ of an area A is defined by the line integral

$$\Psi = \int_{\partial A} \vec{B}(x,y,z) \cdot d\vec{l},$$

where $d\vec{l}$ is the vectorial line element along the boundary curve, which is oriented counterclockwise.

(1) Draw $\vec{F}(x,y,z)$ in the x-y-plane.

(2) Add a rectangle of your choice to your sketch and draw the vectorial line elements as well as the projection of the field components onto the line elements to each of the four sides of the rectangle.

(3) Qualitatively evaluate the contributions of the line integrals over the boundary curve of the rectangle: Is the line integral over the entire boundary curve positive, negative or zero?

(b) Stokes' theorem describes the relationship between the curve of a vector field $\vec{B}(x,y,z)$ within an area A and the line integral over the (closed) boundary curve of the area ∂A,

$$\int_A \operatorname{curl} \vec{B} \cdot d\vec{n} = \int_{\partial A} \vec{B} \cdot d\vec{l}.$$

(1) Give a comprehensible explanation of $\int_A \operatorname{curl} \vec{B} \cdot d\vec{n}$ To do this, use the qualitative interpretation of curl from exercise sheet 3 and of surface integrals.

(2) Using $\vec{F}(x,y,z)$ (in the x-y-plane) as an example, verify the relationship between the change in field components and the circulation around a closed curve. Draw a sketch.

(3) Use the simulation to determine the circulation of $\vec{F}(x,y,z)$ around the boundary curve of a square with an edge length of 1. Using a calculation, show that the result is equal to the left side of Stokes' theorem.

(c) From Stokes' theorem, we get the coordinate-independent form of the curl, where $d\vec{n} = \hat{n}dA$:

$$(\operatorname{curl} \vec{B}) \cdot \hat{n} = \lim_{A\to 0} \frac{1}{A} \int_{\partial A} \vec{B} \cdot d\vec{l}.$$

(1) Use the limit value relation above to explain why the curl of a field can be described as a measure of vorticity at a certain location.

(2) Explain the relationship between the vorticity of a vector field and its path (in)dependence.

(d) Consider an air flow in the x-z-plane. The horizontal velocity v_x of the associated velocity vector field $\vec{v}(x, y=0, z) = v_x(z)\hat{e}_x$ increases with increasing vertical height z.

(1) Draw $\vec{v}(x, y=0, z)$ in the x-z-plane.

(2) Add a rectangle A to your sketch and explain why vortices form in air flows of this kind. How does the size and shape of the inserted form A affect your explanation? Give reasons for your answer.

2.2 Traditional learning tasks

Learning Task: Divergence of a vector field
The divergence of a three-dimensional, differentiable vector field $\vec{A}(\vec{r})$ with $\vec{r} = (x, y, z) \in \mathbb{R}^3$ is defined as follows:

$$\operatorname{div} \vec{A} = \vec{\nabla} \cdot \vec{A} := \frac{\partial}{\partial x} A_x + \frac{\partial}{\partial y} A_y + \frac{\partial}{\partial z} A_z \,.$$

Here $\vec{\nabla}$ indicates the nabla operator in Cartesian coordinates

$$\vec{\nabla} = \frac{\partial}{\partial x} \hat{e}_x + \frac{\partial}{\partial y} \hat{e}_y + \frac{\partial}{\partial z} \hat{e}_z = (\partial_x, \partial_y, \partial_z)^T \,.$$

Similarly, in polar coordinates, the divergence of a two-dimensional vector field $\vec{A}(\vec{r'})$ with $\vec{r'} = \vec{r}(r, \varphi)$ is defined by

$$\operatorname{div} \vec{A} = \frac{1}{r} \frac{\partial}{\partial r} (r A_r) + \frac{1}{r} \frac{\partial}{\partial \varphi} A_\varphi \,.$$

Furthermore, let $r = |\vec{r}| = \sqrt{\vec{r} \cdot \vec{r}}$ be the magnitude of the position vector $\vec{r}$.

(a) Find the divergence of:

(1) $\hat{e}_x$

(2) $\hat{e}_r \in \mathbb{R}^2$

(3) $\vec{r} \in \mathbb{R}^3$

(4) $(x^2 + yz)\, \hat{e}_x + (y^2 + zx)\, \hat{e}_y + (z^2 + xy)\, \hat{e}_z$

(5) $-kr\hat{e}_r$, where $k \in \mathbb{R}$ is constant

(6) $x^2 \hat{e}_x + e^{xy} \hat{e}_y + xyz\hat{e}_z$ at the position $(-1, 1, 2)$

(7) $-k\,(x\hat{e}_x + y\hat{e}_y)$, where $k \in \mathbb{R}$ is constant

(8) $xy\hat{e}_x + \hat{e}_y$

(b) Show that for the partial Cartesian derivatives of a function $f = f(r)$ (with $r = |\vec{r}| = \sqrt{x^2 + y^2 + z^2}$), which only depends on the radius r, the following relationship applies: $\partial_i f(r) = \frac{x_i}{r} \frac{\mathrm{d}f}{\mathrm{d}r}$ $(x_i = x, y, z)$.

(c) Prove the following relations ($\vec{r} = (x, y, z) \in \mathbb{R}^3$):

(1) Show that for any differentiable function $f(r)$ the following applies:

$$\vec{\nabla} \cdot [f(r)\vec{r}] = 3f(r) + rf'(r) \,.$$

(2) Use this to show that $\vec{\nabla} \cdot [f(r)\vec{r}] = 0$ if and only if $f(r) = \text{const}/r^3$ $(r \neq 0)$.

(3) Now set $f(r) = 1/r^m$ $(r \neq 0)$ and look at the result of $\vec{\nabla} \cdot [f(r)\vec{r}]$ for $m = 0$, $m = 1$ and $m = 2$.

Learning task: Gauss' theorem

Consider $\vec{B}(\vec{r})$, where $\vec{r} = (x, y, z) \in \mathbb{R}^3$.

(a) Calculate the surface integral

$$\int_A (2xz\hat{e}_x + (x+2)\hat{e}_y + y(z^2-3)\hat{e}_z) \cdot d\vec{n}$$

for the surface of a cube with an edge length of 2, where $0 \leq x, y, z \leq 2$.

(b) For a volume V, Gauss' theorem is defined as

$$\int_V \operatorname{div} \vec{B}\, dV = \int_{\partial V} \vec{B} \cdot d\vec{n}\,.$$

(1) Verify Gauss' theorem for $\vec{B}(\vec{r}) = \vec{r}$, where the left-hand side is integrated over a sphere with radius R and the right-hand side is integrated over the sphere's surface.

(2) Calculate the flux of $\vec{B}(\vec{r}) = xy\hat{e}_x + 2yz\hat{e}_y + 3zx\hat{e}_z$ through a cube with an edge length of 2, where $-1 \leq x \leq 1$, $-1 \leq y \leq 1$ and $0 \leq z \leq 2$. Use Gauss' theorem to verify your result.

(c) Show that for a scalar function $T(x, y, z)$, the following relation applies:

$$\int_V (\vec{\nabla} T)\, dV = \int_{\partial V} T d\vec{n}$$

Tip: Use Gauss' theorem and $\vec{B} = \vec{c}T$, *where* $\vec{c} \in \mathbb{R}^3$ *is constant.*

Learning task: Curl of a vector field

For a three-dimensional, differentiable vector field $\vec{A}(\vec{r})$, where $\vec{r} = (x, y, z) \in \mathbb{R}^3$, the curl of $\vec{A}$ is defined as follows:

$$\operatorname{curl} \vec{A} = \vec{\nabla} \times \vec{A} := \left(\frac{\partial A_z}{\partial y} - \frac{\partial A_y}{\partial z} \right) \hat{e}_x + \left(\frac{\partial A_x}{\partial z} - \frac{\partial A_z}{\partial x} \right) \hat{e}_y + \left(\frac{\partial A_y}{\partial x} - \frac{\partial A_x}{\partial y} \right) \hat{e}_z \,.$$

Where $\vec{\nabla}$ indicates the nabla operator

$$\vec{\nabla} = \frac{\partial}{\partial x}\hat{e}_x + \frac{\partial}{\partial y}\hat{e}_y + \frac{\partial}{\partial z}\hat{e}_z = (\partial_x, \partial_y, \partial_z)^T \,.$$

Similarly, in cylindrical coordinates, the curl of a three-dimensional vector field $\vec{A}(\vec{r'})$, where $\vec{r'} = \vec{r}(r, \varphi, z)$, is defined as follows:

$$\operatorname{curl} \vec{A} = \left(\frac{1}{r}\frac{\partial A_z}{\partial \varphi} - \frac{\partial A_\varphi}{\partial z} \right) \hat{e}_r + \left(\frac{\partial A_r}{\partial z} - \frac{\partial A_z}{\partial r} \right) \hat{e}_\varphi + \frac{1}{r} \left(\frac{\partial}{\partial r}(r A_\varphi) - \frac{\partial A_r}{\partial \varphi} \right) \hat{e}_z \,.$$

Furthermore, let $r = |\vec{r}| = \sqrt{\vec{r} \cdot \vec{r}}$ be the magnitude of the position vector $\vec{r}$ and $\vec{B}(\vec{r})$ another three-dimensional, differentiable vector field in $\mathbb{R}^3$.

(a) Show that for any differentiable function $f(r)$ the following relation applies at all times: ($\vec{r} = (x, y, z) \in \mathbb{R}^3$): $\vec{\nabla} \times [f(r)\vec{r}] = \vec{0}$.

(b) Find the curl of the following functions:

(1) $\hat{e}_x$

(2) $\hat{e}_r$

(3) $\vec{r}$

(4) $(x^2 + yz)\,\hat{e}_x + (y^2 + zx)\,\hat{e}_y + (z^2 + xy)\,\hat{e}_z$

(5) $\vec{r}(x^2 + y^2 + z^2)^{\pm 3/2}$

(6) $-y\hat{e}_x + x\hat{e}_y$

(7) $\frac{\vec{r}}{r^a}$ $(a > 0,\ \vec{r} \neq \vec{0})$

(8) $\vec{A} = (2xy^2 + 4z^3)\hat{e}_x + 2x^2y\hat{e}_y + 12xz^2\hat{e}_z$ and $\vec{B} = (2xy^2 + 4z^3)\hat{e}_x + 2x^2y\hat{e}_y + (12xz^2 + x^2y^2)\hat{e}_z$
For which field does the curl disappear?

(9) $r\hat{e}_\varphi$

(10) $xy\hat{e}_x + \hat{e}_y$

(c) Prove the following relations:

(1) $\vec{\nabla} \cdot (\vec{\nabla} \times \vec{A}) = 0$

(2) $\operatorname{div}[\vec{A} \times \vec{B}] = \vec{B} \cdot \operatorname{curl} \vec{A} - \vec{A} \cdot \operatorname{curl} \vec{B}$

(3) $\operatorname{curl}[\vec{A} \times \vec{B}] = \vec{A} \operatorname{div} \vec{B} - \vec{B} \operatorname{div} \vec{A} + [\vec{B} \cdot \vec{\nabla}]\vec{A} - [\vec{A} \cdot \vec{\nabla}]\vec{B}$

Learning task: Stokes' theorem

Consider $\vec{B}(\vec{r})$, where $\vec{r} = (x, y, z) \in \mathbb{R}^3$.

(a) Calculate the line integral

$$\int_{C_i} (x^2 y \hat{e}_x - x y^2 \hat{e}_y) \cdot d\vec{l}$$

for two (partially) straight curves C_1 and C_2:

(C_1) from $(0, 0, 0)$ to $(1, 0, 0)$ and then to $(1, 1, 0)$

(C_2) directly from $(0, 0, 0)$ to $(1, 1, 0)$.

(b) For a surface A, Stokes' theorem is defined as

$$\int_A \operatorname{curl} \vec{B} \cdot d\vec{n} = \int_{\partial A} \vec{B} \cdot d\vec{l}.$$

(1) Verify Stokes' theorem for $\vec{B}(\vec{r}) = \vec{r}$, where the left-hand side is integrated over a sphere with radius R and $z \geq 0$ while the right-hand side is integrated over the sphere's surface.

(2) Calculate the line integral of $\vec{B}(\vec{r}) = 6\hat{e}_x + yz^2\hat{e}_y + (3y + z)\hat{e}_z$ along a triangular path in the y-z-plane with the vertices $(0, 0, 0)$, $(0, 1, 0)$, and $(0, 0, 2)$. Verify your answer using Stokes' theorem.

(c) Show that for a scalar function $T(x, y, z)$, the following relation applies:

$$\int_A (\vec{\nabla} T) \times d\vec{n} = -\int_{\partial A} T d\vec{l}$$

Tip: Use Stokes' theorem with $\vec{B} = \vec{c}T$, where $\vec{c} \in \mathbb{R}^3$ is constant.
